# A Cabled Acoustic Telemetry System for Detecting and Tracking Juvenile Salmon: Part 2. Three-Dimensional Tracking and Passage Outcomes

**DOI:** 10.3390/s110605661

**Published:** 2011-05-26

**Authors:** Z. Daniel Deng, Mark A. Weiland, Tao Fu, Tom A. Seim, Brian L. LaMarche, Eric Y. Choi, Thomas J. Carlson, M. Brad Eppard

**Affiliations:** 1 Pacific Northwest National Laboratory, P.O. Box 999, Richland, WA 99332, USA; E-Mails: mark.weiland@pnl.gov (M.A.W.); tao.fu@pnnl.gov (T.F.); Thomas.Seim@pnl.gov (T.A.S.); brian.lamarche@pnl.gov (B.L.L.); eric.choi@pnl.gov (E.Y.C.); thomas.carlson@pnl.gov (T.J.C.); 2 U.S. Army Corps of Engineers, Portland District, P.O. Box 2946, Portland, OR 97208, USA; E-Mail: matthew.b.eppard@usace.army.mil

**Keywords:** acoustic tracking, underwater acoustic sensors, acoustic telemetry

## Abstract

In Part 1 of this paper, we presented the engineering design and instrumentation of the Juvenile Salmon Acoustic Telemetry System (JSATS) cabled system, a nonproprietary sensing technology developed by the U.S. Army Corps of Engineers, Portland District (Oregon, USA) to meet the needs for monitoring the survival of juvenile salmonids through the hydroelectric facilities within the Federal Columbia River Power System. Here in Part 2, we describe how the JSATS cabled system was employed as a reference sensor network for detecting and tracking juvenile salmon. Time-of-arrival data for valid detections on four hydrophones were used to solve for the three-dimensional (3D) position of fish surgically implanted with JSATS acoustic transmitters. Validation tests demonstrated high accuracy of 3D tracking up to 100 m upstream from the John Day Dam spillway. The along-dam component, used for assigning the route of fish passage, had the highest accuracy; the median errors ranged from 0.02 to 0.22 m, and root mean square errors ranged from 0.07 to 0.56 m at distances up to 100 m. For the 2008 case study at John Day Dam, the range for 3D tracking was more than 100 m upstream of the dam face where hydrophones were deployed, and detection and tracking probabilities of fish tagged with JSATS acoustic transmitters were higher than 98%. JSATS cabled systems have been successfully deployed on several major dams to acquire information for salmon protection and for development of more “fish-friendly” hydroelectric facilities.

## 1. Introduction

Despite advances in turbine design and dam operations, passage through turbines and spillways may injure or kill downstream-migrating juvenile salmon [1–6]. The design and operation of more fish-friendly hydroelectric facilities require reliable estimates of behavior, timing, and survival of the juvenile salmonids as they migrate downstream [7–10]. Three-dimensional (3D) position estimates of fish surgically implanted with acoustic transmitters can provide near-dam fish behavior and passage route-specific survival rates. In addition, 3D position estimates provide near-dam vertical distribution data required for other important turbine passage evaluation techniques such as blade-strike modeling [5,10].

Position estimation algorithms are used to locate a moving or stationary object using a reference sensor network. Different signals and sensors are selected depending on applications. The Global Positioning System (GPS) uses satellites to provide location estimates by measuring the time of arrival (TOA) of radio signals [11,12]. An underwater acoustic tracking system provides reliable location information of the sources by measuring TOA of acoustic pulses or calls from aquatic animals using a network of underwater hydrophones or receivers with known locations [13,14]. However, the basic principles underlying the two systems are the same except for different requirements for the TOA measurement and geometric configuration of the sensor network.

Underwater acoustic tracking has become a common technology for studying and monitoring the movement and behavior of aquatic animals [15,16]. Usually the absolute time required for the acoustic signal to travel from the source location to the hydrophone is unknown, so time of arrival differences (TOADs) are computed. For 3D source location estimation, a network of at least four hydrophones is required so that four unknown variables—the reference TOA and the three coordinates of the source location—can be solved using four quadratic (nonlinear) distance equations. When one component of the source location is derived from another method, such as a pressure or depth sensor, the minimum number of hydrophones required is then reduced to three. For two-dimensional (2D) source location, at least three hydrophones are required because there are three unknown variables [17]. When there are more hydrophones than the minimum requirement (*i.e.*, with redundant hydrophones), this problem becomes an over-determined system [17,18].

Many researchers have investigated this nonlinear problem, and several solvers have been developed mathematically for different applications [13–29]. Watkins and Schevill [13] first described the 3D position estimate problem using four hydrophones and developed a geometric method. Exact solutions in various forms were also discussed by Fang [22], Wahlberg *et al.* [15], Spiesberger and Fristrup [14], and Bucher and Misra [28]. However, an exact solution may not always be available due to the nonlinearity of the four distance equations, errors in TOA or TOAD measurements, errors in sound speed, and hydrophone location uncertainties. In such cases, it is necessary to consider it as an optimization problem and estimate the source location by minimizing the errors.

The most common approximation employs iterative Taylor-series methods or variant Newton-Gaussian methods, which linearize the equation using Taylor expansion and search for an approximate numerical solution iteratively by minimizing the least-square error [20]. Foy [20] produced accurate position estimates at reasonable signal-to-noise ratios (SNRs). However, the solution was very sensitive to the initial conditions. Chan and Ho [25] introduced an intermediate variable to transform the nonlinear distance equations into linear equations containing the new intermediate variable and the original unknown variables. The authors then used an approximate realization of the maximum likelihood estimator to find a noniterative and explicit solution and compared their results with those from iterative Taylor-series methods. They found improved accuracy and efficiency at relatively high SNR and small TOAD estimation errors. Wahlberg *et al.* [15] synthesized the methods proposed by Watkins and Schevill [13] and Spiesberger and Fristrup [14] and developed a general mathematical form for 2D and 3D systems and for both minimum number of receivers arrays and over-determined arrays. Au and Herzing [16] successfully tracked dolphins using a star geometry in which a four-receiver array was arranged as a symmetrical star. Chan *et al.* [30] improved their maximum likelihood (ML) algorithms by starting from ML functions instead of linearizing the equations first, then derived a closed-form approximate maximum likelihood algorithm. The authors demonstrated the new method’s superior performance in 2D experiments.

In Part 1 of this paper [31], we presented the engineering design and instrumentation of the Juvenile Salmon Acoustic Telemetry System (JSATS) cabled system, a nonproprietary sensing technology developed by the U.S. Army Corps of Engineers, Portland District (OR, USA). All hydrophones are synchronized to the universal GPS clock using a GPS card (Model GPS170 PCI, Meinberg Funkuhren GmbH & Co. KG, Bad Pymont, Germany), resulting in detection time accuracy on a single system to 250 ns and across multiple systems to 500 ns. In addition, all JSATS components are required to pass comprehensive acceptance and performance tests in a controlled environment before they are deployed in the field [31,32]. Part 2 of this paper describes how the JSATS cabled system was employed and evaluated in the field as a reference sensor network for detecting and tracking juvenile salmon.

The efficiencies of exact solvers are approximately 90% possibly because of the high accuracy of clock management, hydrophone location survey, and improved performance of JSATS components. Therefore, only the performance of exact solvers is presented in this paper.

## 2. Methods

### 2.1. Study Site

**Figure 1.**
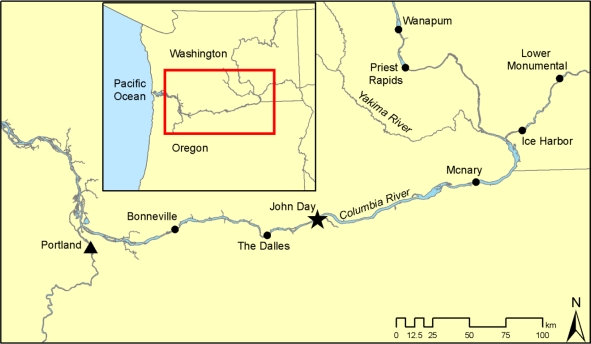
Location of John Day Dam on the Columbia River at rkm 348.

The John Day Dam, owned and operated by the U.S. Army Corps of Engineers, is located on the Columbia River at river kilometer (rkm) 348, approximately 45 km east of the city of The Dalles, Oregon (Figure 1). The dam consists of a powerhouse, spillway, and navigation lock, with fish ladders at either end of the dam. The powerhouse is 602 m long and consists of 16 turbines with nameplate capacity of 135 MW each and overload capacity of 155.3 MW each. The spillway has an overall length of 374 m and contains 20 gates, each 15.2 m wide. Two prototype top-spill weirs (TSWs) were installed at spillbays 15 and 16 during 2008.

### 2.2. Algorithm

Consider a transmitting source in a four-hydrophone array. Throughout this section, the boldface letters indicate matrices or vectors. The source (S) and receiver (r) position vectors are defined as:

(1) $$\begin{array}{cc} S={\left(s_{x},\;s_{y},\;s_{z}\right)}^{\text{T}} & \\ r_{i}={\left(x_{i},\;y_{i},\;z_{i}\right)}^{\text{T}} & i\;=\;0,1,2,3 \end{array}$$

The distance between transmitting source and hydrophones gives:

(2) $${\left(s_{x}\;-\;x_{i}\right)}^{2}\;+\;{\left(s_{y}\;-\;y_{i}\right)}^{2}\;+\;{\left(s_{z}\;-\;z_{i}\right)}^{2}\;=\;c^{2}\;{\left(t_{i}\;+\;T_{0}\right)}^{2},\;\;\;\;\;i\;=\;0,1,2,3$$

where c is the speed of sound, *T*_0_ is the time of travel from the source to the reference receiver (receiver 0), and *t_i_* is the TOAD between receiver i and the reference receiver. With *t_i_* measured by the common clock, the source position vector and *T*_0_ are the four unknowns.

Assuming the first receiver is located at the origin of the coordinate system and subtracting Equation (2) for i = 0 from Equation (2) for i = 1, 2, and 3 [14,15], we obtain:

(3) $$2R^{T}\;S\;+\;2c^{2}tT_{0}\;=\;b$$

where:

(3a) $$R\;=\;\left[\begin{array}{ccc} x_{1} & x_{2} & x_{3}\\ y_{1} & y_{2} & y_{3}\\ z_{1} & z_{2} & z_{3} \end{array}\right],\;t\;=\;\left(\begin{array}{c} t_{1}\\ t_{2}\\ t_{3} \end{array}\right),\;b\;=\;\left(\begin{array}{c} b_{1}\\ b_{2}\\ b_{3} \end{array}\right),\;\text{and}\;b_{i}\;=\;{||r_{i}||}^{2}\;-\;c^{2}t_{i}^{2}$$

From Equation (3):

(4) $$S\;=\;R^{-T}\;(\frac{1}{2}b\;-\;c^{2}tT_{0})$$

Substituting Equation (4) into $S^{T}S\;=\;c^{2}T_{0}^{2}$ gives:

(5) $$T_{0}\;=\;\frac{-p\;\pm \;\sqrt{p^{2}\;-\;aq}}{a}$$

(5a) $$a\;=\;c^{4}t^{T}\;R^{-1}R^{-T}t\;-\;c^{2},\;p\;=\;-\frac{1}{2}c^{2}t^{T}\;R^{-1}R^{-T}b,\;\text{and}\;q\;=\;\frac{1}{4}b^{T}R^{-1}R^{-T}b$$

Note that there are two possible solutions for *T*_0_. If both are complex, there is no solution for the given configuration and TOADs. A negative *T*_0_ is not possible. When there are two real non-negative solutions, then both provide possible locations for the source. It is then necessary to identify which one is physically possible. After *T*_0_ is determined, the source position (S) is obtained by Equation (4).

For this study, all hydrophones were installed at the dam face and looking upstream into the dam forebay. In addition, the hydrophones were baffled with plastic cones lined with an anechoic material to exclude loud noises emanating from structures downstream of the hydrophones. Therefore, the physical solution from two real non-negative solutions would be upstream of the dam. When more than four hydrophones detected the same transmitter message, the four with the optimum geometric configuration for 3D tracking were selected using the criteria developed by Wahlberg *et al.* [15] and Ehrenberg and Steig [33].

After the source location was obtained from 3D tracking, TOADs (*t*′_1_, *t*′_2_, *t*′_3_) and *T*′_0_ were computed using the estimated source location for the given hydrophone locations and speed of sound. Speed of sound was calculated using an equation developed by Marczak [34]. The total time error was then defined as:

$$\Delta T\;=\;\sqrt{{\left({\text{t}}_{1}^{\prime }\;-\;{\text{t}}_{1}\right)}^{2}\;+\;{\left({\text{t}}_{2}^{\prime }\;-\;{\text{t}}_{2}\right)}^{2}\;+\;{\left({\text{t}}_{3}^{\prime }\;-\;{\text{t}}_{3}\right)}^{2}\;+\;{\left({\text{T}}_{0}^{\prime }\;-\;{\text{T}}_{0}\right)}^{2}}$$

The detailed steps for 3D tracking and passage outcomes for this study are as follows:

1. Pool all detections of the same signal from different hydrophones. If more than four hydrophones detect the same tag signal, select the four with the best geometric configuration for 3D tracking. Compute the TOAD directly from detection time because all hydrophones are synchronized to a universal GPS clock with accuracy within 0.5 μs.
2. Apply tracking solvers to estimate 3D locations and output solutions that are physical and within the prespecified Δ*T* (10 μs in this study).
3. Apply order 3 median filtering [35] to remove spurious locations and smooth fish tracks. Assign a route of passage based on the along-dam component of the last tracked location.
4. Assign another set of passage routes based on the detections of the last two hydrophones at different piers. For example, if the two hydrophones were at Pier 1 (numbering starting from the Oregon side) and Pier 2, then the passage route would be assigned to the first turbine unit.
5. Compare the two sets of passage routes. If the difference for a fish is more than one bay, check its trajectory and detection history manually.

### 2.3. Validation and Test Cases

**Figure 2.**
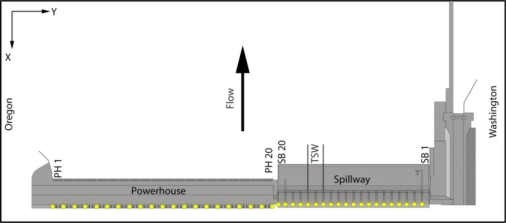
Location of JSATS hydrophones on the dam face of John Day Dam. The yellow dots represent hydrophone locations.

To assess the accuracy of the deployed hydrophone arrays and validate tracking solvers, several tests were conducted using acoustic transmitters with GPS receivers fixed at various locations or drifting upstream of the John Day Dam powerhouse (PH) and spillway. Two hydrophones were installed at each main pier nose at two elevations throughout the dam (Figure 2).

All of the systems had similar functional and geometric designs, so only one spillbay (SB) was selected for model validation and error analysis. The locations of the acoustic transmitters for all test cases were obtained through a real-time kinematic GPS system (Trimble RTK 5700 with Zephyr Geodetic antenna, Trimble Navigation Ltd., Sunnyvale, CA, USA), which provided benchmark measurements for comparison with the 3D-tracked locations. The accuracy was assessed in terms of median and root mean square (RMS) values of the differences between GPS measurements and the source locations computed from 3D tracking codes:

$$\Delta x_{i}\;=\;|x_{i}^{3D}\;-\;x_{i}^{GPS}|,\;\;\;\;\;i\;=\;1,\ldots N$$

$$\Delta y_{i}\;=\;|y_{i}^{3D}\;-\;y_{i}^{GPS}|,\;\;\;\;\;i\;=\;1,\ldots N$$

$$\Delta z_{i}\;=\;|z_{i}^{3D}\;-\;z_{i}^{GPS}|,\;\;\;\;\;i\;=\;1,\ldots N$$

$$\Delta d_{i}\;=\sqrt{\Delta x_{i}^{2}\;+\;\Delta y_{i}^{2}\;+\;\Delta z_{i}^{2}},\;\;\;\;\;i\;=\;1,\ldots N$$

$${RMS}_{x}\;=\;\sqrt{\frac{1}{N}\sum_{i=1}^{N}\Delta x_{i}^{2}}$$

$${RMS}_{y}\;=\;\sqrt{\frac{1}{N}\sum_{i=1}^{N}\Delta y_{i}^{2}}$$

$${RMS}_{z}\;=\;\sqrt{\frac{1}{N}\sum_{i=1}^{N}\Delta z_{i}^{2}}$$

$${RMS}_{d}\;=\;\sqrt{\frac{1}{N}\sum_{i=1}^{N}\Delta d_{i}^{2}}$$

where *N* was the number of estimated positions and *x*, *y*, and *z* were the three components in the dam-face coordinate system. The dam-face coordinate system was defined as follows: the *x*-axis was perpendicular to the dam and looking straight into the forebay; the *y*-axis was along the dam face from the Oregon to the Washington side; and the *z*-axis was vertical, pointing upward.

**Figure 3.**
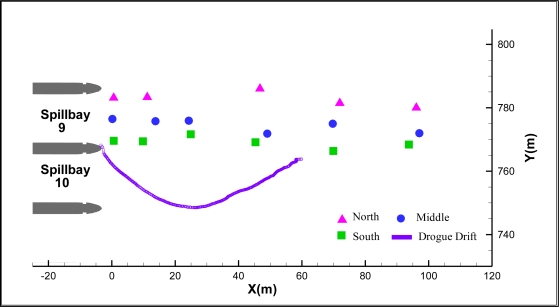
Error analysis test locations at John Day Dam spillway.

The acoustic transmitters used in the validation tests had a ping rate of one pulse per second and a source level of 155 dB relative to 1 μPa at 1 m. Transmitters were attached at different water depths to a rope held steady by an anchor at the bottom. For the fixed-location tests, seven transmitters were suspended at 1, 2, 3, 5, 10, 15, and 20 m below the water surface and held at various locations from 5 m to 100 m upstream of the dam in the forebay (Figure 3). Each fixed location test lasted approximately 10 min, resulting in a typical sample size of 550 transmissions, given the 1-s pulse rate repetition. For the drogue drift, six transmitters were held at 1, 2, 3, 5, 10, and 15 m below the water surface. The GPS measurement point was about 1 m above the water surface. Because of the windy conditions and underwater currents, the rope holding the transmitters was not always steady, resulting in large uncertainties in transmitter locations in deep water. For this reason, only the transmitter held at 2 m below water surface was employed for the accuracy assessment, although all transmitters were included for tracking efficiency analysis.

### 2.4. Field-Scale Application at John Day Dam

This case study involved a total of 2,445 yearling Chinook salmon (YC) and 2,448 steelhead (STH) in Spring and in 2,483 subyearling Chinook salmon (SYC) in Summer passing through John Day Dam during 2008. Median lengths of tagged fish for these downstream migrating YC, STH, and SYC were 158 mm, 217 mm, and 117 mm, respectively. All fish were surgically implanted [36] with JSATS acoustic transmitters and passive integrated transponder (PIT) tags [37]. The size of the JSATS acoustic transmitters differed from spring to summer due to technological advances in transmitter design. In Spring, the transmitter mean weight was 0.485 g in air and 0.324 g in water, and transmitters were nominally 12.46 mm long, 5.30 mm wide, and 3.70 mm high. In Summer, the tag mean weight was 0.425 g in air and 0.290 g in water. Summer tags averaged 12.04 mm long, 5.27 mm wide, and 3.74 mm high. The acoustic transmitters used in this study had a ping rate of one pulse every 3 s to provide an expected transmitter life of at least 23 days.

Tagged YC and STH were released daily over a 29-day period in Spring (May 1 through May 29) near Arlington, OR, USA at rkm 390 (42 km upstream of John Day Dam, at 0,600, 1,200, and 1,800 hours). Similarly, acoustic-tagged SYC were released in summer over a 29-day period (June 15 through July 13) in three release groups at Arlington, OR, USA (at 0,600, 1,200, and 2,100 hours).

To receive signals from tagged fish, we deployed shallow and deep JSATS cabled hydrophones on the upstream face of John Day Dam with a total 21 systems and 84 hydrophones (dam-face array). We also deployed and maintained autonomous node arrays [38] at river cross sections, including 2 km upstream of John Day Dam (forebay array) and 9.4 km downstream of the dam (tailwater egress array). The forebay array was used to create a virtual release for fish as they enter the forebay 2 km upstream of the dam. The dam-face array was used to create a virtual release for fish known to have passed John Day Dam and to estimate route of passage at the dam using 3D tracking and last-detection data. The time of last detection by the dam-face array minus the time of first detection on the forebay array provided an estimate of forebay residence time. The time of first detection by the John Day Dam tailwater egress array minus the time of last detection on the dam-face array provided an estimate of relative egress time. The PIT-tag detection system was used to provide the percentage of fish passing the PH that were guided into the juvenile bypass facilities at John Day Dam.

Fish passage and behavior at the dam relative to the TSWs and two spill treatments (30% *versus* 40% spill out of total water discharge through the dam) were investigated using detections at the dam face coupled with forebay hydrophone detections and acoustic tracking. Fish detections were classified into “arrival blocks” based on forebay array data and “passage blocks” based on dam-face array data. The blocks corresponded to areas of the dam, moving from south to north, as indicated in Figure 2: PH turbine units 1–8, units 9–16, skeleton bays 17–20, SBs 17–20, TSW bays 15–16, and SBs 1–14. Skeleton bays were included in arrival blocks but not in passage blocks because fish could not pass there.

## 3. Results and Discussion

### 3.1. Validation Results

Tracking efficiency was evaluated as the number of successful 3D-tracked locations divided by the number of transmissions. All transmitters had high tracking efficiencies at distances of 5 m to 100 m from the dam face (Table 1). The transmitter attached to the rope at 20 m below the surface broke off before the 5-m location test. Occasionally, there were low tracking efficiencies at a few locations; for example, Transmitter 4 had an efficiency of 54.9% at 5 m and Transmitter 3 had an efficiency of 66.8% at 100 m. Tag 1 had lower efficiencies likely because of the multipath from the surface. These infrequent dips in tracking efficiency were likely due to the fact that we could not control the directivity of the transmitters during the tests.

Only the results of Transmitter 2 were selected for accuracy assessment. The median errors of the *x-*component (the distance to the dam face) ranged from 0.06 to 0.55 m at distances up to 75 m and ranged from 0.52 to 1.18 m at 100 m distance (Table 2).

**Figure 4.**
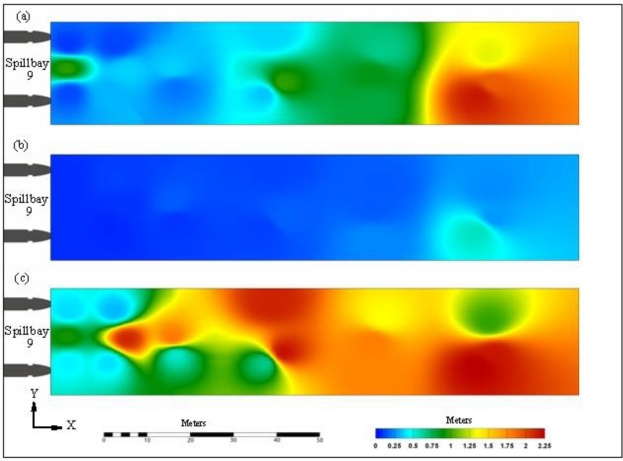
Contour plots of RMS errors of the transmitter at 2 m below the water surface, John Day Dam spillway: **(a)** *x*; **(b)** *y*, **(c)** *z*.

The RMS errors fell between 0.1 and 0.98 m for distances up to 75 m and between 1.31 and 2.16 at 100 m distance. Figure 4 contains interpolation of the Transmitter 2 results. The *y*-component, used for the route assignment, had the highest accuracy among the three components. The median errors at SB 9 ranged from 0.02 to 0.22 m, and RMS errors ranged from 0.07 to 0.56 m at distances up to 100 m. When the distance was less than 50 m, the median errors and RMS errors were within 0.07 and 0.16 m, respectively. The *z*-component was in the vertical plane. At SB 9, the median errors of z-component ranged from 0.31 to 0.87 m, and the RMS errors ranged from 0.33 to 2.25 m for all distances. For absolute distances, the median errors were within 1 m for all distances except at the 100-m distance in the south and middle section (1.64 m and 1.67 m, respectively). RMS errors of absolute distances ranged from 0.37 to 3.17 m.

**Figure 5.**
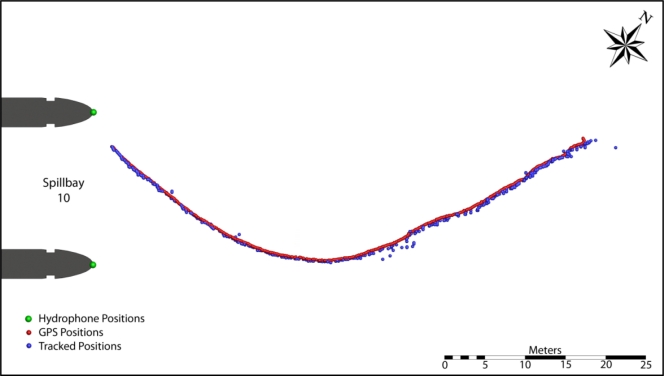
Comparison of GPS measurements and 3D-tracked positions at John Day Dam spillway.

For the drogue drift test, the overall tracking efficiency was 93.2% (Figure 5). The median errors in *x*, *y*, *z*, and total distance were 0.20 m, 0.22 m, 0.38 m, and 0.52 m, respectively. *RMS_x_*, *RMS_y_*, *RMS_z_*, and *RMS_d_* were 0.49 m, 0.27 m, 0.95 m, and 1.10 m, respectively.

Both the median and RMS errors were computed from 3D-tracked positions that were slightly smoothed by order 3 median filtering without removing outliers. If outliers were removed, or if additional smoothing such as Kalman filtering algorithms were applied, the RMS errors could be reduced significantly. In addition, windy conditions and underwater current probably caused inaccuracies between GPS measurements and true transmitter locations, which would result in an increase in RMS errors.

### 3.2. Case Study

**Figure 6.**
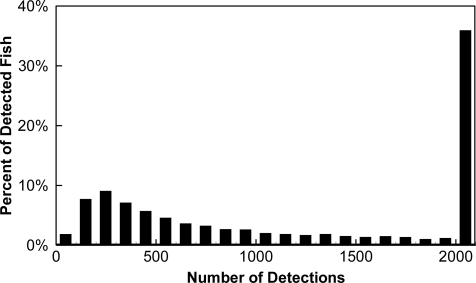
Number of detections by cabled systems at John Day Dam, 2008.

**Figure 7.**
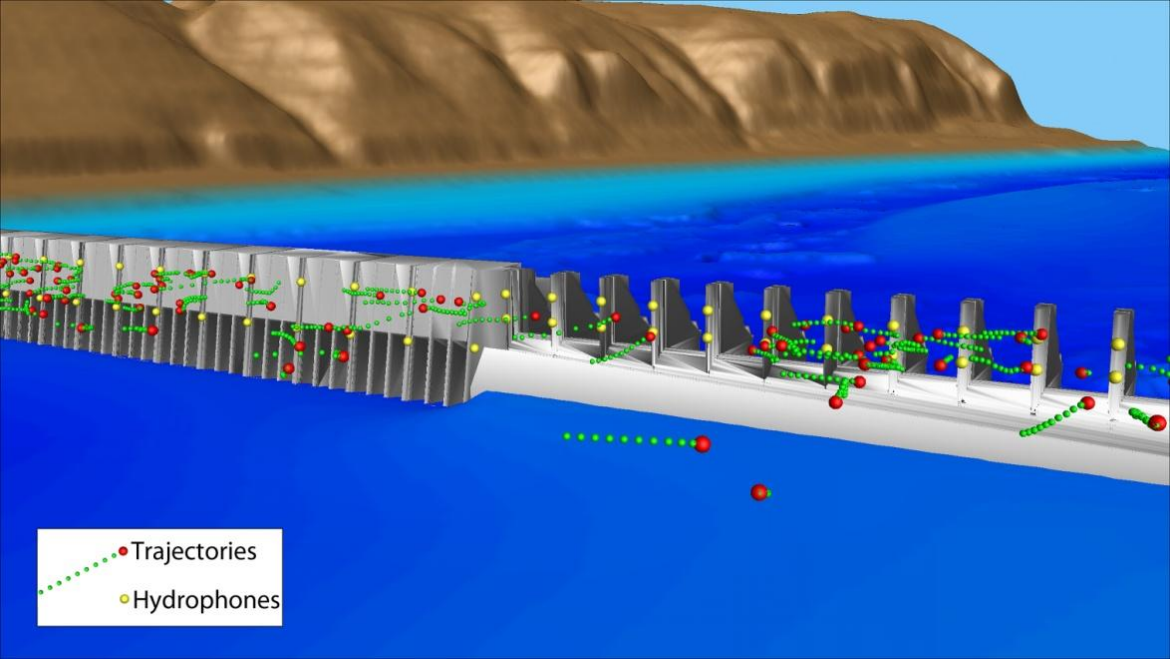
Examples of 3D tracks at John Day Dam, 2008. Note that water surface is not plotted.

Of the 7,376 tagged fish released upstream of John Day Dam, 7,118 (97%) were detected by the autonomous receivers in the forebay. Of these 7,118 tagged fish, 7,067 (99%) were detected by the cabled systems, with a median number of detections of 1,066 (Figure 6). Another 6,975 (98%) were 3D-tracked; the median number of tracking positions was 101 (Figure 7).

**Figure 8.**
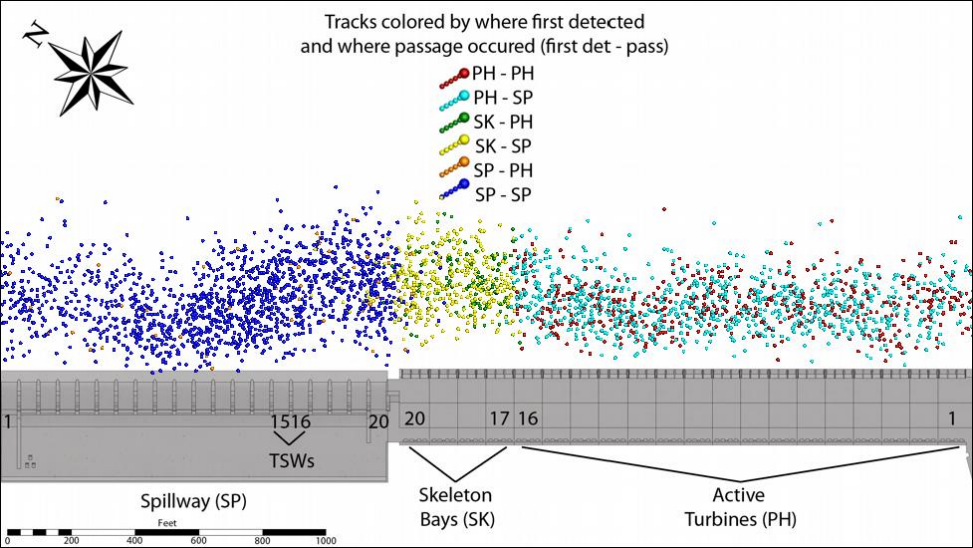
Distribution of passage sections and first detection of yearling Chinook salmon at John Day Dam, 2008.

At least half of the tagged fish arriving upstream of the PH turbine units and skeleton bays moved north to ultimately pass at the spillway, including the TSWs. This pattern was strongest for STH and weakest for SYC. Tagged fish arriving upstream of the spillway, however, did not tend to move south toward the PH (Figure 8). Specifically, for YC arriving in the forebay, 45% and 16% approached the PH upstream of turbine units 1–16 and the skeleton bays, respectively, whereas 12% arrived at SBs 17–20, 5% at the TSWs, and 22% at SBs 1–14. Of the fish first arriving at the PH, nearly 60% moved north and passed at the spillway, mostly at SBs 17–20 and the TSWs, closer to the PH. Conversely, 2% of YC arriving at the spillway moved south and passed via the PH. The remaining 98% passed via the spillway or TSWs.

For acoustic-tagged STH arriving in the forebay, 52% and 12% approached the PH upstream of turbine units 1–16 and the skeleton bays, respectively. Arrivals at the spillway included about 9% at SBs 17–20, 4% at the TSWs, and 23% at SBs 1–14. Nearly 66% of the STH that arrived at the PH moved north and passed at the spillway, primarily at the TSWs. Similar to YC, the majority of STH first approaching the spillway typically passed at SBs 1–14 or the TSWs; few STH arriving at the spillway moved south and passed at the PH. Overall, a noticeable portion of STH moved toward the TSWs, regardless of arrival block. For SYC, about 60% of those detected in the forebay arrived upstream of the PH turbine units and the skeleton bays. About half of these fish moved north to ultimately pass at the spillway, mostly at SBs 17–20 and the TSWs. Of the 25% of total SYC arriving at units 9–16, more than one-half passed there or at units 1–8 and did not move north toward the spillway.

Vertical distribution data were based on 3D tracking of individual acoustic-tagged fish. As smolts moved from 75 m to within 10 m of the PH face, travel depths often decreased, but there was a sudden increase to more than 20 m at a distance of less than 5 m from the PH. Note that PH piers on which hydrophones were mounted do not extend more than about 1 m upstream of the PH face so that sounding fish within 5 m of the dam face can be tracked moving down toward intake openings. The turbine intake ceilings at John Day Dam are about 20 m deep. At the spillway, detection depths were less than 5 m, regardless of distance upstream from the face of the spillway.

There was no difference in diel vertical distributions for PH- or spillway-passed YC. For STH, vertical distribution was shallow for fish passed through the spillway; this pattern was especially evident at the TSW. The last-detection depths at the PH were much deeper. Most SYC in the forebay of the PH and skeleton bays traveled at depths between 5 and 11 m, while median depths of smolts within 5 m of the PH or skeleton bays were between 20 and 25 m. As with YC and STH, the last-detection depths for SYC were relatively shallow at the spillway. Notably, as SYC approached the TSW, they migrated upward in the water column, but this trend was not evident for their approach at non-TSW SBs.

## 4. Conclusions

Time-of-arrival information for valid detections on four hydrophones was used to solve for the 3D position of fish surgically implanted with JSATS acoustic transmitters. Validation tests demonstrated high accuracy of 3D tracking up to a 100-m distance at the John Day Dam spillway. The along-dam component used for assigning the route of fish passage had the highest accuracy; the median errors ranged from 0.02 to 0.22 m, and RMS errors ranged from 0.07 to 0.56 m at distances up to 100 m. For the case study at John Day Dam during 2008, the range for 3D tracking was more than 100 m upstream of the dam face, where hydrophones were deployed and detection and tracking probabilities were more than 98%. JSATS cabled systems have been successfully deployed on several major dams to acquire information for salmon protection and for development of more “fish-friendly” hydroelectric facilities.

## Figures and Tables

**Table 1.** Tracking efficiency of JSATS cabled system at fixed locations.

**Transmitter index**	**Depth from surface (m)**	**Tracking efficiency (%) at**					
		**5 m**	**15 m**	**30 m**	**50 m**	**75 m**	**100 m**
1	1	87.0	89.0	88.6	86.8	69.3	49.9
2	2	93.9	95.5	88.8	96.7	87.4	80.3
3	3	92.1	89.1	90.4	93.0	86.7	66.8
4	5	54.9	99.6	99.1	98.9	94.8	86.8
5	10	98.8	99.3	99.6	98.8	98.2	96.5
6	15	84.6	99.5	98.6	99.1	98.3	97.7
7	20	N/A	89.1	98.7	98.5	99.1	95.8

**Table 2.** Median and RMS errors of the transmitter at 2 m below the water surface at SB 9, John Day Dam spillway.

**Location**	**Distance (m)**	**Median (Δx_i_)**	**Median (Δy_i_)**	**Median (Δz_i_)**	**Median(Δd_i_)**	**RMS_x_**	**RMS_y_**	**RMS_z_**	**RMS_d_**
**North**	5	0.06	0.06	0.39	0.40	0.10	0.08	0.39	0.41
	15	0.06	0.07	0.35	0.37	0.12	0.11	0.33	0.37
	50	0.22	0.07	0.57	0.64	0.38	0.12	2.13	2.16
	75	0.40	0.11	0.46	0.68	0.66	0.16	1.35	1.51
	100	0.52	0.13	0.42	0.78	1.31	0.25	0.99	1.66

**Middle**	5	0.34	0.05	0.31	0.69	0.97	0.08	0.93	1.35
	15	0.06	0.05	0.41	0.44	0.37	0.09	2.11	2.15
	30	0.21	0.02	0.87	0.94	0.37	0.16	1.82	1.86
	50	0.34	0.11	0.53	0.71	0.98	0.17	2.19	2.41
	75	0.51	0.11	0.58	0.99	0.86	0.16	1.80	2.00
	100	1.18	0.17	0.59	1.64	1.92	0.27	2.17	2.90

**South**	5	0.07	0.06	0.40	0.42	0.13	0.08	0.43	0.45
	15	0.06	0.04	0.40	0.40	0.32	0.07	0.40	0.52
	30	0.11	0.05	0.53	0.56	0.21	0.09	0.54	0.59
	50	0.18	0.04	0.56	0.60	0.35	0.11	0.66	0.75
	75	0.55	0.18	0.57	0.95	0.79	0.24	1.78	1.96
	100	1.13	0.22	0.59	1.67	2.16	0.56	2.25	3.17
